# The Modern Treatment of Syphilis

**Published:** 1907-04-27

**Authors:** D'Arcy Power

**Affiliations:** Surgeon to and Lecturer on Surgery at St. Bartholomew's Hospital


### Hospital Clinics.

### THE MODERN TREATMENT OF SYPHILIS.

By D'ARCY POWER, M.A., M.B. Oxon., F.R.C.S. Eng., Surgeon to and Lecturer on Surgery at St. Bartholomew's Hospital.

For all practical purposes syphilis appeared in Europe as a new disease in 1494, though it had long been recognised in the New World. The Spaniards who sailed with Columbus in his first voyage became infected, and introduced it into the Old World at a convenient opportunity for its wide distribution. John of Vigo (1460-1517), writing as a contemporary, says: "In the yeare of our Lord 1494, in the monethe of December, when Charles the French kynge tok hys jorney into the partes of Ytaly, to recouer the kyngdome of Naples, there appeared a certayne disease throughout all Ytaly of an unknowen nature, which sondry nations hath called by sondry names. The Frenchemen call it the dysease of Naples, bycause the souldyers brought it from thence into Fraunce. The Neapoloitanes call it Frenche dysease, for it appered fyrste when they came to Naples, and so other languages call it by other names." In the end it became known as Morbus Gallicus, which the common people shortened to Morbus. The now familiar name of Syphilis was given to it, or at any rate the word was rendered familiar, by the widely read poem "Syphilis, sive Morbus Gallicus," written by Jerome Fracastoro, who was born at Verona about 1483. The first mention of the word occurs in the following lines:—

> Syphilus (ut fama est) ipsa haec ad flumina pastor
> Mille boves, niveas mille haec per pabula regi
> Alcithoo pascebat oves.

(The story runs that Syphilus, a herdsman, tended by these streams a thousand oxen, and in these meads fed a thousand snow-white sheep all belonging to King Alcithous.) It is remarkable that no medical writer before 1500 even hinted that syphilis had any connection with "the syne of venerie," though the fact was shrewdly suspected by the Town Council of Aberdeen as early as April 21st, 1497, on which day "it was statut and ordanit by the Alderman and Council for the eschewin of the infirmitey cumm out of France and strange partis that all licht wemen be chargit and ordanit to decist fra their vices under the payne of ane key of het yron on their cheekis and banysene of the toune."

### Syphilis in the Middle Ages.

It was partly owing to this ignorance of its origin and partly from the lax morality prevalent at the end of the fifteenth century that no great moral turpitude attached to the disease on its first appearance. Benvenuto Cellini, the Florentine goldsmith (1500-1570) says that "in Rome this kind of illness is very partial to the priests, and especially the richest of them." He tells us that it was at first treated in the native American manner with guaiacum, and that the methods of fumigation and inunction with mercury were originally employed at Rome by Giacomo Berengario da Carpi. These methods had no great success. He only undertook a cure after stipulating for fees which he reckoned not by tens but by hundreds of crowns. "He was a person of great sagacity," says Cellini, "and did wisely to get out of Rome, for not many months afterwards all the patients he had treated grew so ill that they were a hundred times worse off than before he came. He would certainly have been murdered if he had stopped." His method consequently fell into disrepute, and, speaking in another part of his autobiography, Cellini says "that Charlatan Maestro Jacopo, the surgeon from Carpi, came to Rome and spent six months there, during which he bedaubed some scores of noblemen and unfortunate gentlefolk with his dirty salves, extracting many thousands of ducats from their pockets, and at the present moment in Rome all the miserable people who used his ointment are crippled, and in a deplorable state of health."

From the time of Berengario until the present day mercury has been used in the cure of syphilis, at first empirically, because syphilis was thought to be allied to certain skin diseases for which mercurial ointments had been prescribed; afterwards blindly, because it had been the custom to treat syphilis with mercury; only recently with discretion, because it is recognised that by mercury alone can syphilis really be cured.

It seems presumptuous at first sight to speak of the modern treatment of syphilis when one has nothing but mercury to offer, and mercury has been used for at least four hundred years in every part of the civilised world where syphilis has been present. But mercury has been abused both in the amount given, in the method of administration, and in the concomitants and adjuvants, until it fell into such disrepute that there are still many persons who have inherited an instinctive horror of the drug. When mercury was given in the earlier days the patient was first prepared by bleeding, enemata, frequent bathing, prohibition of wine and all nourishing food. He was then shut up in a closed room, the windows of which were never opened during the cure, the room being heated and the mercury rubbed in whilst the patient stood before a roaring fire. After each inunction the parts were covered with tow or wool and the patient was put into a warmed bed and covered with thick bedclothes to make him sweat freely. The cure lasted from twenty to thirty days, and during this time the linen was left unchanged, lest any of the mercury should be lost. Very little food was given, soups, beef-tea, the yolks of eggs, and rice, forming the staple, whilst quantities of drugs were given to evacuate the evil humours. Even as late as 1753 Boerhaave, the great Dutch physician, ordered enemata every four hours. The effect of this treatment was to cause severe salivation, and it is marvellous that any of the patients recovered. "A good salivation" was one in which the patient produced five or six pounds of viscid saliva in twentyfour hours, and this salivation was sometimes maintained for thirty or forty days. During the last hundred years these auxiliaries to treatment have one by one disappeared. There is still perhaps faith in diet, and many hold it as an article of belief that syphilis cannot be cured unless the gums "be touched," as it is called, or, in other words, unless the patient has been salivated. But they have thought so little about the matter that when once this point has been reached they are satisfied; and, because there has been some stomatitis, and the patient has been cured of his present symptoms, he is allowed to leave off any further treatment.

Emphasis is now beginning to be laid upon a feature of syphilis which has long been known—namely, that it is a disease of very long duration, marked by periods of remission and periods of exacerbation. There is a slight tendency towards spontaneous cure under the most favourable conditions of health in any given patient, but in the majority of cases the disease merely lies dormant, ready to manifest itself on every occasion when the general health becomes undermined or in any way reduced, whether by accident or from such natural causes as senile changes advancing more rapidly in one organ than another. There is very little doubt that over-exertion of a part or of a tissue will render it more liable to syphilitic inflammation than if it be maintained in a normal condition. It is further recognised that in the great majority of cases syphilis can be cured so completely that the affected parent will be capable of producing healthy children.

### The Prophylaxis of Syphilis.

The present knowledge of syphilis teaches that every patient must have an initial lesion at the seat of inoculation, and that for a short time it is a local disease. During this period it can be attacked at the seat of lesion and the disease arrested; but it quickly becomes generalised, and is then beyond the reach of topical remedies.

Dr. Paul Maisonneuve, working with Metchnikoff and Roux in Paris, has recently published an interesting thesis on the prophylaxis of syphilis, in which he shows that, as in the case of vaccination, the inoculation of syphilis is effected better through a scarification or slight superficial wound than through one which involves the deeper layers of the skin. At first syphilis remains as a local infection, and it can then be treated locally, just as a poisoned wound can be disinfected immediately after it has been produced. The experiments on apes and a chimpanzee show that the interval between the inoculation and absorption of syphilis is short, but measurable; and if mercurial applications be made during this interval, the further manifestation of the disease can be prevented, for the poison is thereby destroyed. Indeed, so certain are the French pathologists of their power to arrest the general infection of the body by local means that they allowed Dr. Maisonneuve to be inoculated with syphilis. At the end of an hour the inoculated places were treated with an ointment of calomel, and no further signs of syphilis appeared, whilst the control animals who were inoculated simultaneously developed chancres in due course. Although the syphilitic infection in this particular experiment was arrested in an hour to avoid any chance of the disease developing, there is reason to suppose that the local application of a mercurial ointment will hinder syphilis as long as eighteen hours after inoculation. If such a result is verified by further experiments, the knowledge will prove of the greatest service to those who are likely to contract the disease in the course of their professional work, and such people are dentists, doctors, and midwives. The ointment employed is made by rubbing up mercury and lanolin in the proportion of 1 to 10 by weight, and the ointment must be rubbed into the inoculated place for at least ten minutes.

### Treatment of Early Syphilis.

These experiments dispense at once with the bad system of waiting, in cases of syphilis, until the appearance of secondary symptoms "clears up the diagnosis." When the primary sore appears it is too late to act on the syphilitic poison by any local means, for by this time it has become generalised; but mercury destroys the virus or its cause, and the sooner it is given, the more likely is the patient to be relieved of further symptoms. Hitherto it has been withheld, because it was thought necessary to give large doses and to carry the effects to an extreme. So far as is known scientifically, small doses alone are required, but they must be continued for long periods of time. The salivation of patients during a mercurial course for the treatment of syphilis should be considered as great a reflection on a surgeon as the occurrence of suppuration in one of his operation cases.

The modern treatment of syphilis aims at treating the disease and not merely its symptoms. Yet, in spite of this knowledge, English practitioners are nearly all "opportunists," that is to say, they treat the symptoms as they arise, but when once the rash, the mucous patch, the sore throat, or the falling hair has been cured, they are content to let the patient go untreated until the appearance of fresh signs compel him to seek advice again.

The proper and only means of curing syphilis is a prolonged course of mercury from the time the patient is first seen until the time of the secondary manifestations is well over, and for empirical purposes this is taken to be one year from the period of infection. The patient may occasionally have a rest from treatment, and the form in which the mercury is given may be changed. Personally, I prefer the administration of grey powder for private patients, and the injection of Colonel Lambkin's mercurial cream for hospital patients. When mercury is given by the mouth it is stopped for three days at the end of each month and for a fortnight at the end of every third month, tonics or cod-liver oil and maltine being taken in the intervals. It is often necessary, too, to suspend the administration or reduce the dose for a day or two on account of trifling digestive disorders; but so long as the weight of the patient is maintained and he appears to be improving in general health, the dose of mercury should be steadily continued.

The simpler the preparation of mercury used the more satisfactory are the results in the treatment of syphilis. Hardly a week passes in which some new mercurial compound is not brought into notice with a flourish of trumpets worthy of Andrew Boorde or the other sixteenth century quacksalvers. The fundamental error in connection with these preparations is that they are mere mixtures, and are not chemical compounds, so that there is no certainty of their composition being identical at different times, or with different samples. The prescriber does not know, therefore, what quantity of mercury he is giving, nor in what form.

### Treatment of Late Syphilis.

The administration of iodide of potassium is too often relied upon exclusively for the treatment of tertiary or gummatous syphilis. It is, indeed, excellent, but it should be remembered that it only removes the products of syphilitic inflammation in the same manner as it removes the products of other chronic inflammations, whether they be rheumatic, cancerous, or actinomycotic in origin. Mercury will produce the same results as iodide of potassium in the tertiary lesions of syphilis, but mercury acts much more slowly. It is, however, curative, and the appearance of gummatous inflammation in syphilis shows that the disease is still active, though in a chronic and perhaps attenuated form. Logically, therefore, a course of treatment by iodides should always be followed by, or associated with, the administration of mercury. Too much must not be expected of the iodides apart from their action on newly formed inflammatory tissue. They possess the power of acting upon the mononuclear leucocytes, which are so numerous in syphilis, and thus cause the absorption of the new and imperfect connective tissue so long as it is cellular in character; but they have no action on fully formed fibrous or scar tissue. But the very word "absorption" is merely used for convenience, since no explanation is yet forthcoming about the manner in which the iodides cause the disappearance of newly formed connective tissue.

It was formerly the practice to give five or ten grain doses of potassium iodide three times a day for many months. It is now usual to give larger doses of fifteen to thirty grains at a time for a week, and then to replace it by a tonic mixture containing iron, nux vomica, and dilute phosphoric acid for another week, the iodide being again given as before. The body is thus prevented from becoming used to the circulation of iodides through the tissues, and better results are obtained in the treatment of tertiary syphilis. The larger doses of iodide are conveniently given dissolved in some effervescing mineral water at meal times.

The modern treatment of syphilis has resolved itself into a cure of the disease by the use of mercury in its crudest form and stripped of all the adjuncts which were formerly thought to be essential to its success. This change of plan is the result of an extended study of experimental pathology which has shown the part played by micro-organisms in the production of disease. Syphilis, by all analogy, ought to be caused by a micro-organism, and some of the best pathologists of the present day believe that the micro-organism has been already discovered in a particular variety of spirochæte named after its discoverer the spirochæta of Schaudinn, or more specifically the *Spirochæta pallida*, an actively moving protozoon, which differs from the *Spirochæta refringens*, a coarser form, which is not uncommonly found on the mucous membranes of healthy persons. It is too soon, as yet, to make any dogmatic statement, but evidence is accumulating daily to show that the secret of the syphilitic virus is at length discovered.

